# Efficacy of standard and low dose hydrochlorothiazide in the recurrence prevention of calcium nephrolithiasis (NOSTONE trial): protocol for a randomized double-blind placebo-controlled trial

**DOI:** 10.1186/s12882-018-1144-6

**Published:** 2018-12-10

**Authors:** Nasser A. Dhayat, Nicolas Faller, Olivier Bonny, Nilufar Mohebbi, Alexander Ritter, Lisa Pellegrini, Giulia Bedino, Carlo Schönholzer, Reto M. Venzin, Carina Hüsler, Irene Koneth, Rosaria Del Giorno, Luca Gabutti, Patrizia Amico, Michael Mayr, Urs Odermatt, Florian Buchkremer, Thomas Ernandez, Catherine Stoermann-Chopard, Daniel Teta, Felix Rintelen, Marie Roumet, Irina Irincheeva, Sven Trelle, Luca Tamò, Beat Roth, Bruno Vogt, Daniel G. Fuster

**Affiliations:** 1 Department of Nephrology and Hypertension, Inselspital, Bern University Hospital, University of Bern, Bern, Switzerland; 2 Department of Nephrology, CHUV, University Hospital Lausanne, University of Lausanne, Lausanne, Switzerland; 3 Department of Nephrology, University Hospital Zurich, Zürich, Switzerland; 4 Department of Nephrology, Regional Hospital Lugano, Lugano, Switzerland; 5 Department of Nephrology, Cantonal Hospital Chur, Chur, Switzerland; 6 Department of Nephrology and Transplantation Medicine, Cantonal Hospital St. Gallen, St. Gallen, Switzerland; 7 Department of Nephrology, Regional Hospital Bellinzona, Bellinzona, Switzerland; 8 Medical Outpatient Department, University Hospital Basel, University of Basel, Basel, Switzerland; 9 Department of Nephrology, Cantonal Hospital Luzern, Luzern, Switzerland; 10 Division of Nephrology, Dialysis and Transplantation, Cantonal Hospital Aarau, Aarau, Switzerland; 11 Department of Nephrology, HUG, University Hospital Geneva, University of Geneva, Geneva, Switzerland; 12 Service de Nephrology, Centre Hospitalier du Valais Romand (CHVR), Sion, Switzerland; 13 Clinical Trials Unit, University of Bern, Bern, Switzerland; 14 Department of Urology, Inselspital, Bern University Hospital, University of Bern, Bern, Switzerland

**Keywords:** Nephrolithiasis, Kidney stones, Recurrence, Prevention, Hydrochlorothiazide

## Abstract

**Background:**

Nephrolithiasis is a global healthcare problem with a current lifetime risk of 18.8% in men and 9.4% in women. Given the high cost of medical treatments and surgical interventions as well as the morbidity related to symptomatic stone disease, medical prophylaxis for stone recurrence is an attractive approach. Thiazide diuretics have been the cornerstone of pharmacologic metaphylaxis for more than 40 years. However, evidence for benefits and harms of thiazides in the prevention of calcium containing kidney stones in general remains unclear. In addition, the efficacy of the currently employed low dose thiazide regimens to prevent stone recurrence is not known.

**Methods:**

The NOSTONE trial is an investigator-initiated 3-year prospective, multicenter, double-blind, placebo-controlled trial to assess the efficacy of standard and low dose hydrochlorothiazide treatment in the recurrence prevention of calcium containing kidney stones. We plan to include 416 adult (≥ 18 years) patients with recurrent (≥ 2 stone episodes in the last 10 years) calcium containing kidney stones (containing ≥50% of calcium oxalate, calcium phosphate or a mixture of both). Patients will be randomly allocated to 50 mg or 25 mg or 12.5 mg hydrochlorothiazide or placebo.

The primary outcome will be incidence of stone recurrence (a composite of symptomatic or radiologic recurrence). Secondary outcomes will be individual components of the composite primary outcome, safety and tolerability of hydrochlorothiazide treatment, changes in urinary biochemistry elicited by hydrochlorothiazide treatment and impact of baseline disease severity, biochemical abnormalities and stone composition on treatment response.

**Discussion:**

The NOSTONE study will provide long-sought information on the efficacy of hydrochlorothiazide in the recurrence prevention of calcium containing kidney stones. Strengths of the study include the randomized, double-blind and placebo-controlled design, the large amount of patients studied, the employment of high sensitivity and high specificity imaging and the exclusive public funding support.

**Trial registration:**

ClinicalTrials.gov, NCT03057431. Registered on February 20 2017.

## Background

Nephrolithiasis is a worldwide healthcare problem with a current lifetime risk of ~ 18.8% in men and ~ 9.4% in women in Western civilizations [1]. Incidence and prevalence of renal stone disease are increasing globally, irrespective of age, sex and race [1, 2]. Without a specific treatment, 5- and 20-year recurrence rates are ~ 40% and ~ 75%, respectively [3, 4]. In the United States, hospitalizations, surgery and lost work time associated with kidney stones cost more than 5 billion US Dollars annually [5]. Thus, given the high cost and the morbidity related to recurrent kidney stone disease, medical prophylaxis seems to be an attractive approach [6, 7]. Indeed, apart from its benefits to patients in terms of reduced morbidity and risk from procedures, medical prevention of nephrolithiasis is clearly cost effective [8].

Eighty to 90% of stones are composed of calcium oxalate, calcium phosphate or a mixture of both [9, 10]. Increased excretion of calcium in the urine, hypercalciuria, is the most frequent metabolic abnormality encountered in patients with recurrent nephrolithiasis [10, 11]. The hypercalciuria encountered in recurrent stone formers is often familial and strongly influenced by diet, but in most cases of unknown origin and hence designated “idiopathic” [3]. Gut absorption of calcium is enhanced in idiopathic hypercalciuria, but serum calcium remains typically normal because intestinally absorbed calcium is promptly excreted by the kidneys [12]. Despite intestinal calcium hyperabsorption, patients with idiopathic hypercalciuria are often in negative calcium balance because of excessive renal calcium losses, especially under a low calcium diet [13, 14]. As a consequence, low bone mass is a frequent finding in normo- and especially hypercalciuric stone formers [15]. Thiazide diuretics are the only drugs known to reduce urinary calcium excretion. This peculiar property is employed in the prevention of recurrent calcium nephrolithiasis but also in the prevention of bone loss in patients with recurrent nephrolithiasis and/or arterial hypertension [15–20].

The efficacy of thiazides in the recurrence prevention of kidney stones has been studied in several randomized controlled trials (RCTs) (Table 1) [21–31]. With the exception of two trials [29, 31], thiazides significantly reduced stone recurrence compared to placebo or control. However, as detailed in Table 1 and highlighted by a recent systematic review [32], thiazide RCTs thus far conducted suffer from significant methodological deficiencies, including: use of high thiazide doses, low overall number of patients studied, lack of outcome uniformity, use of outdated dietary recommendations, unclear allocation concealment, lack of double-blinding and intention-to-treat analysis, absence of adverse event and drop out reporting, unknown baseline risk of disease severity and baseline biochemical abnormalities of patients studied, lack of patient stratification and employment of low sensitivity and specificity imaging modalities.

**Table 1:** Randomized controlled trials of thiazides in the prevention of recurrent nephrolithiasis

Author, Year	Treatment, Dose	Allocation Concealment	Blinding	Intention-to-treat Analysis	Withdrawals described	Selection for Hypercalciuria	Follow-Up (Years)	Treated/ Placebo, n/N	Events/Total, n/N Thiazide	Events/Total, n/N Placebo	RR ^c^	Recurrence Outcome
Brocks, 1981 [29]	Bendroflumethiazide, 2.5 mg TID ^a^	Unclear	Double-blind	No	No	No	1.6	33/29	5/33	5/29	NS	Composite
Scholz, 1982 [31]	HCTZ, 25 mg BID ^b^	Unclear	Double-blind	No	No	No	1	25/26	6/25	6/26	NS	Symptomatic
Laerum, 1984 [23]	HCTZ, 25 mg BID	Unclear	Double-blind	Yes	Yes	No	3	23/25	5/23	12/25	0.45	Composite
Wilson, 1984 [26]	HCTZ, 100 mg daily	Unclear	Open-label	No	No	No	2.8	23/21	0.15 stones/year	0.32 stones/year	0.48	Symptomatic
Robertson, 1985 [27]	Bendroflumethazide, 2.5 mg TID	Unclear	Open-label	No	No	No	3–5	13/9	0.22 stones/year	0.58 stones/year	0.38	Symptomatic
Mortensen, 1986 [24]	Bendroflumethazide, 2.5 mg	Unclear	Double-blind	No	No	No	2	12/10	0/12	4/10	–	Composite
Ettinger, 1988 [22]	Chlorthalidone, 25 m /50 mg	Adequate	Double-blind	No	Yes	No	3	19/23/31 (25 mg /50 mg/placebo)	6/42	14/31	0.32	Composite
Ohkawa, 1992 [25]	Trichlormethiazide, 4 mg	Unclear	Open-label	No	No	Yes	2.14–2.21	82/93	24/82	57/93	0.42	Composite
Borghi, 1993 [21]	Indapamide, 2.5 mg daily	Unclear	Open-label	No	Yes	Yes	3	25/25	3/25	9/25	0.33	Composite
Ahlstrand, 1996 [30]	HCTZ, 25 mg BID	Unclear	Open-label	Yes	Yes	Yes	3.6–4.3	17/22	9/17	19/22	0.61	Composite
Fernandez-Rodriguez, 2006 [28]	HCTZ, 50 mg daily	Unclear	None stated	Yes	No withdrawals	No	3	50/50	16/50	28/50	0.57	Composite

^a^TID, three times daily, ^b^ BID, twice times daily, ^c^ RR, relative risk

Hydrochlorothiazide (HCTZ) was used in five of the 11 thiazide RCTs for stone prevention and is thus currently the best studied thiazide in the prevention of stone recurrence [23, 26, 28, 30, 31]. However, other thiazides such as bendroflumethiazide, chlorthalidone, trichlormethiazide and indapamide also reduced stone recurrence in one or more trials and seem to be effective as well. In all trials, high thiazide doses were employed, in the case of HCTZ, 50–100 mg daily. In four of the five HCTZ trials, the diuretic was given twice daily, whereas in the treatment of arterial hypertension, HCTZ is typically given once daily [33]. Once daily HCTZ at the dose of 50 mg, 25 mg or 12.5 mg reduces calciuria in healthy volunteers, a surrogate marker for stone prevention [34]. Detailed HCTZ dose-response studies with respect to urinary composition and stone recurrence are lacking. Twice daily HCTZ increases the frequency of side effects and augments diuresis at night and thereby likely affects compliance [33, 34].

A recent study revealed that thiazide diuretics are often not used in an evidence-based fashion for the prevention of stone recurrence [35]. The tendency to prescribe lower doses of thiazides in patients with recurrent nephrolithiasis was likely triggered by a paradigm shift in prescribing practices for thiazides used for the treatment of arterial hypertension. Starting in the 1980’s, lower doses of HCTZ (12.5–25 mg daily) were increasingly employed [36]. While clinical and biochemical side effects were noted to be dose-dependent, the antihypertensive effects remained robust, even at lower doses [36, 37]. In the case of recurrent nephrolithiasis, however, this practice is not supported by randomized evidence and consequently, we do not know whether the currently employed low dose thiazide regimens are effective in reducing the risk for stone recurrence.

Thus, evidence for benefits and harms of thiazides in the prevention of calcium containing kidney stones *in general* remains unclear. In addition, the efficacy of the currently employed low dose thiazide regimens to prevent stone recurrence is not known.

## Methods / design

### Study objectives

#### Overall objective

The NOSTONE study aims to describe an efficacy and safety profile of HCTZ for the recurrence prevention of calcium nephrolithiasis.

#### Primary objective

Dose-response relationship for three different dosages of HCTZ using incidence of stone recurrence (a composite of symptomatic or radiologic recurrence) as the primary outcome.

#### Secondary objectives

Efficacy of the different dosages of HCTZ in terms of the primary outcome as well as the individual components of the composite primary outcome, i.e. incidence of symptomatic stone recurrence and incidence of radiologic stone recurrence. Effects of different dosages of HCTZ on urinary biochemistry (efficacy and safety aspects) and the impact of different baseline characteristics on the effects of the different dosages (effect modification).

#### Safety objectives

Long-term safety and tolerability of HCTZ compared to placebo.

### Study outcomes

#### Primary outcome

The primary outcome of the NOSTONE study is the incidence of stone recurrences during study treatment. Stone recurrence is the composite of symptomatic or radiological recurrences. Symptomatic stone recurrence is defined as visible passage of a stone or typical symptoms such as colicky flank/loin pain with hematuria or any stone (symptomatic or asymptomatic) requiring urological intervention for stone removal. Radiological stone recurrence as assessed by low-dose non-intravenous contrast CT imaging is defined as the appearance of new calculi or enlargement of preexisting calculi with reference to the baseline CT performed at randomization.

#### Secondary outcomes

(i) The individual components of the composite primary outcome, i.e. number of symptomatic stone recurrences and number of radiologic stone recurrences.

(ii) Changes in urinary biochemistry elicited by HCTZ or placebo.

(iii) Impact of baseline disease severity (incidence of stone recurrence during the last 10 years  prior to randomization) and biochemical abnormalities on stone recurrence.

(iv) Impact of stone composition on stone recurrence.

#### Safety outcomes

Safety endpoints to be analyzed include a descriptive summary of the following parameters:

i) SAEs.
ii) Pre-specified AEs of special interest including:
  Hypokalemia, defined as blood potassium level < 3 mmol/L.
  Hyponatremia, defined as blood sodium level < 125 mmol/L.
  Hypomagnesemia, defined as blood magnesium level < 0.5 mmol/L.
  Gouty arthritis if recurrence > 3 times per year or requiring uric acid lowering therapy.
  Newly developed overt diabetes mellitus (defined as fasting glucose level ≥ 7 mmol/L or random Glucose ≥11 mmol/L or hemoglobin A1c ≥ 6.5%).
  Allergic reaction of skin if considered by the local investigator to be potentially related to the study medication.
iii) Vital signs.
  Heart rate, systolic and diastolic blood pressure at the right arm in sitting position after at least 5 min rest will be recorded at all study visits.

### Study design

**Fig. 1:**
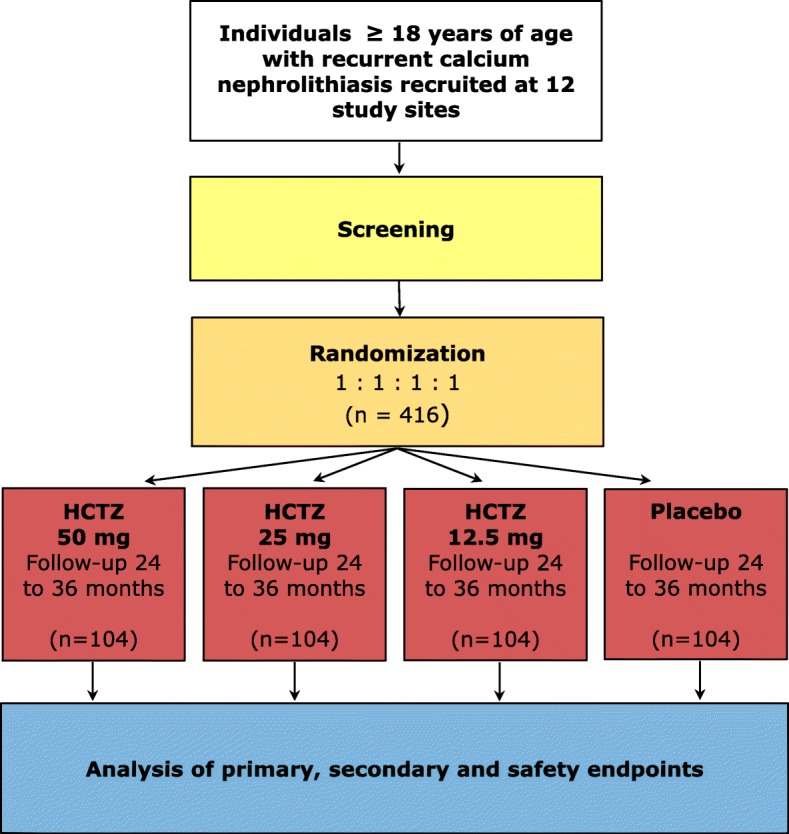
Flow diagram of the NOSTONE trial

The NOSTONE study is an investigator-initiated, randomized, multicenter, double-blind, placebo-controlled phase III trial in which 416 participants will be randomized to four parallel groups (104 participants per group) to receive HCTZ 12.5 / 25 mg / 50 mg or placebo. All subjects will be given the investigational medicinal product (IMP - HCTZ or placebo) once daily in the morning (Fig. 1). Placebo will be administered to individuals randomized to that treatment group in a form identical to the HCTZ capsules. The first IMP dose will be administered the day after the randomization.

Randomization lists are generated by a statistician at CTU Bern, the Clinical Trials Unit of the University of Bern, otherwise not involved in the trial, following dedicated standard operating procedures. Moreover, participants are stratified at randomization according to the number of stone episodes in the last 10 years. Participants with two or three stone episode are clustered in the first stratification group, participants with four or more stones are allocated to a second stratification group. All participants will receive state-of-the-art non-pharmacologic recommendations for stone prevention according to current American [38] and European [16] nephrolithiasis guidelines including: increased fluid intake with circadian drinking to ensure daily urinary volumes of at least 2–2.5 L, a balanced diet rich in vegetables and fibers with normal calcium content (1–1.2 g/day) but limited sodium chloride (4–6 g/day) and animal protein (0.8–1 g/kg/day) content. Furthermore, participants will be advised to retain a normal BMI, have adequate physical activity and balance excessive fluid loss. Participants are followed for a minimum of 24 months and a maximum of 36 months.

#### Study sites

The study is performed at 12 Departments of Nephrology sites throughout Switzerland including seven Cantonal Hospitals (Aarau, Bellinzona, Chur, Lugano, Luzern, Sion, St. Gallen) and five University Hospitals (Basel, Bern, Geneva, Lausanne, Zürich).

### Study population

#### Eligibility criteria

Participants will be recruited according to the eligibility criteria detailed in Table 2.

**Table 2:** Eligibility criteria of the NOSTONE study

Inclusion criteria	
Individuals fulfilling all of the following inclusion criteria are eligible for study participation:	
- Informed Consent as documented by signature	
- Age 18 years or older	
- Recurrent kidney stone disease (≥ 2 stone events within the last 10 years prior to randomization)	
- Any past kidney stone containing 50% or more of calcium oxalate, calcium phosphate or a mixture of both	
Exclusion criteria	
The presence of any one of the following exclusion criteria will lead to exclusion of the individual:	
- Pharmacologic prevention for stone recurrence less than 3 months prior to randomization	
- Patients with secondary causes of recurrent calcareous nephrolithiasis including:	
- Severe eating disorders (anorexia or bulimia)	
- Chronic inflammatory bowel disease, bariatric surgery, intestinal surgery with malabsorbtion or chronic diarrhea	
- Sarcoidosis	
- Primary hyperparathyroidism	
- Complete distal tubular acidosis	
- Active malignancy	
- Patients with the following medications:	
- Thiazide or loop diuretics	
- Carbonic anhydrase inhibitors (including topiramate)	
- Xanthine oxidase inhibitors (febuxostat or allopurinol)	
- Alkali, including potassium citrate or sodium bicarbonate	
- 1,25 -OH Vitamin D (calcitriol)	
- Calcium supplementation	
- Bisphosphonates	
- Denusomab	
- Teriparatide	
- Glucocorticoids	
- Obstructive uropathy, if not treated successfully	
- Urinary tract infection, if not treated successfully	
- Chronic kidney disease (defined as CKD -EPI eGFR < 30 mL/min per 1,73 m^2^ body surface area)	
- Patients with a kidney transplant	
- > 3 gout arthritis episodes within one year prior to randomisation or gout arthritis requiring uric acid lowering therapy	
- Cystinuria at screening	
- Hypokalemia (blood potassium level < 3 mmol/L) at screening	
- Hyponatremia (blood sodium level < 125 mmol/L) at screening	
- Pregnant and lactating women	
- Previous (within 3 months prior to randomization) or concomitant participation in another interventional clinical trial	
- Inability to understand and follow the protocol	
- Known allergy to the study drug	

#### Criteria for withdrawal / discontinuation of participants

Criteria of IMP discontinuation or study discontinuation are listed in Table 3. Participants who permanently discontinue the IMP are expected to continue in the follow-up period and to attend all protocol-specified study visits. If study visits are not possible, a telephone consultation will be performed to determine if relevant health events / endpoints have occurred. A study participant who discontinues study participation prematurely for any reason is defined as dropout if the participant has already been randomized. A study participant who terminates the study before randomization is regarded as a screening failure.

**Table 3:** Criteria for withdrawal / discontinuation of participants

Discontinuation of study investigational medicinal product	
Study IMP must be permanently discontinued if any of the following occurs:	
- If any exclusion criterion applies during the trial, except the incompatible medications. The IMP will be discontinued only if the patient took the medications listed in the exclusion criteria for more than 4 months	
- If the responsible study investigator feels that treatment with the study regimen is harmful to the participant’s well-being	
- If patient is non-compliant with the study intervention as judged by the investigator and/or the sponsor	
- Pregnancy in a study participant	
- Hypokalemia (blood potassium level < 3 mmol/L) not responsive to supplementation therapy	
- Profound hyponatremia (blood sodium level < 125 mmol/L) recurring after temporary suspension of IMP	
- CKD-EPI eGFR < 30 mL/min per 1,73 m^2^ body surface area for more than 3 months	
- Gouty arthritis recurring > 3 times per year or requiring uric acid lowering therapy	
- Allergic reaction of skin as judged by the investigator	
- > 3 recurrences of symptomatic stone events during the trial	
Discontinuation of study	
Study participants must be withdrawn from the study if the following occurs:	
- At the participants own request	
- If, in the investigator’s opinion, continuation of the study would be harmful to the subject’s wellbeing	

#### Study assessments

Outpatients referred to stone clinics for metabolic stone work-up will be recruited for the trial. For work-up and follow-up visits of participants, the NOSTONE protocol strictly adheres to recommendations of the American and European guidelines on nephrolithiasis with regard to scheduling of patient visits, lab analyses and imaging [16, 38]. Prior to randomization, patients will undergo a screening visit to check health status (including lab values), eligibility and determine stone history. At randomization, eligible patients will undergo a low-dose, renal-limited non-iv contrast CT and receive the assigned IMP. Participants enrolled in the trial will be followed-up 3 months after randomization and thereafter with yearly visits and every 3 months through phone calls. Symptomatic recurrence will be assessed at follow-up visits and during phone calls between visits. Radiologic recurrence will be assessed at treatment end by a low-dose, renal-limited non-iv contrast CT.

### Investigational medicinal product (IMP)

HCTZ (ATC code: C03AA03) is one of the best studied thiazides on the market. Thiazides inhibit the sodium/ chloride co-transporter (NCC or SLC12A3) in the distal tubule of the kidney. Inhibition of NCC causes an increased excretion of sodium, chloride and water in the urine, thereby lowering blood pressure. At the same time, thiazides reduce renal calcium excretion by a still ill-defined intrarenal mechanism. In Switzerland, HCTZ as monosubstance is marketed exclusively as Esidrex® by Medius AG, CH-4132 Muttenz, Switzerland in divisible tablets of 25 mg. The approved indications include: arterial hypertension, edemas, heart failure and recurrence prevention of calcium-containing kidney stones. For the treatment of arterial hypertension, Esidrex® is recommended in doses of 12.5–50 mg, once or twice daily. For the recurrence prevention of calcareous nephrolithiasis, Esidrex® is recommended in doses of 25 or 50 mg twice daily. In addition to the monosubstance, HCTZ is currently available in 75 different galenic formulations in Switzerland as combination with ACE-inhibitors, angiotensin II receptor blockers or non-thiazide diuretics (www.swissmedicinfo.ch, last accessed on 01.09.2018). The encapsulated IMP will be provided by Laboratorium Dr. G. Bichsel AG, Interlaken, Switzerland.

### Statistical methods

#### Sample size

Sample size calculation was based on the primary objective i.e. to assess the dose-response relationship and the primary outcome i.e. recurrence with the following assumptions: (i) uniform recruitment over 24 months with allocation ratio fixed at 1 across all arms; (ii) a maximum and minimum follow-up time of 36 and 24 months, respectively; (iii) cumulative drop-out rate of 10% at 24 months after study start; (iv) risk of recurrence in the placebo group of 0.20 and 0.45 at 12 and 36 months after study start, respectively; (v) hazard ratios for the 12.5, 25 and 50 mg HCTZ doses of 0.90, 0.65 and 0.50, respectively; (vi) power was set to be at least 80% and alpha was fixed at a two-sided level of 0.05; (vii) an unweighted log-rank test for linear trend with local alternatives.

#### Statistical analysis

The statistical analysis of the trial will be done at CTU Bern by a statistician first blinded to the group allocation. Blinding will remain in place until the statistician codes the primary analysis of the primary and secondary outcomes and produces a dummy report of the primary analysis using a randomly generated group variable. The true group variable becomes open after the completion of the dummy report and gives place to the final report of all the analysis as well as the quality control by the independent statistician. Primary analyses will be done using the full analysis set according to the intention-to-treat principle where all randomized patients will be analyzed in the allocated group regardless of any protocol violations or early treatment discontinuations. In the secondary per-protocol analysis, we will evaluate primary and secondary outcomes based on the per-protocol analysis set (i.e. considering only subjects who effectively followed the protocol). No formal interim analysis is planned.

#### Primary analysis

We will assess the time to the stone event using the log-rank test stratified for the number of stones at baseline, Kaplan-Meier curves stratified by treatment dose and hazard ratios between dosage groups using the Cox proportional hazards. Comparisons between placebo and the three active trial arms will be considered exploratory, as the trial is not powered to detect differences with placebo. Components of the primary outcome will be analyzed as the primary outcome. Secondary outcomes (changes in urinary biochemistry from baseline and through the study) will be analyzed using the random effects model.

#### Secondary analysis

We will assess the impact of baseline disease severity on stone recurrence; the impact of biochemical abnormalities on stone recurrence; and the impact of stone composition on stone using the multivariable Cox-model .

#### Sensitivity analysis and some additional analyses

We will compare the full analysis set and per protocol analysis of continuous outcomes based on multiple imputations with the analysis of all available cases. We will assess the sensitivity of time-to- stone-event approach comparing it with multiple event models or frailty (count) models or marginal count models. In the secondary analysis in case of a relevant number of patients with multiple events, we will consider a shared-frailty Cox model for multiple recurrent events.

### Quality assurance and control

#### Monitoring

Sites are monitored centrally and by on-site visits by trained monitors of CTU Bern following ICH-GCP guidelines. Trial sites are also regularly visited by the study coordinator and the study sponsor to ensure compliance with study protocol and ICH-GCP guidelines. Complete source data verification (SDV) will be performed by independent monitors.

#### Data management

All the data collected during the trial are stored in a secure electronic data capture system (secuTrial®) according to ICH-GCP guidelines. Secure backup is guaranteed by the University of Bern.

### Study organization

A steering committee oversaw the study design and overviews the conduct of the study. The steering committee is assisted by an advisory committee made up of three experts of international renown in the field of clinical studies or kidney stone disease. A central study coordinator coordinates the study. CTU Bern monitors study progress and quality and completeness of study data.

## Conclusions

Kidney stones belong to the most frequent human diseases and constitute a global health problem. Kidney stones are extremely painful, relapse frequently and cause enormous health care expenditures and excess morbidity. Thus, a well-tolerated, inexpensive and effective approach to prevent kidney stones is highly desired. While thiazides have been the cornerstone of pharmacologic metaphylaxis for several decades, evidence for benefits and harms of thiazides *in general* and *dose-response relationship in particular* in the recurrence prevention of kidney stones remain unclear.

Strengths of the NOSTONE study include the large number of patients studied, the prospective multicenter, parallel-arm, double-blind and placebo-controlled design with stratification by disease activity, the clear allocation concealment and intention-to-treat analysis, the employment of high sensitivity and high specificity imaging, the use of state-of-the-art dietary recommendations, the careful assessment of putative side effects in the stone population and the exclusive public funding support.

The results of the NOSTONE trial will provide patients and physicians alike with long-sought evidence to adapt and hopefully improve preventive measures for calcium containing kidney stones.

## Abbreviations

ACE: Angiotensin converting enzyme
AE: Adverse event
CKD-EPI: Chronic Kidney Disease Epidemiology Collaboration
CT: Computed tomography
CTU: Clinical Trials Unit
GCP: Good Clinical Practice
HCTZ: Hydrochlorothiazide
ICH: International Council for Harmonisation
IICT: Investigator Initiated Clinical Trial
IMP: Investigational medicinal product
NCC: Sodium/chloride co-transporter
RCT: Randomized controlled trial
SAE: Serious adverse event
SDV: Source data verification

## References

[1] Scales CD, Smith AC, Hanley JM, Saigal CS (2012). Prevalence of kidney stones in the United States. Eur Urol.

[2] Romero V, Akpinar H, Assimos DG (2010). Kidney stones: a global picture of prevalence, incidence, and associated risk factors. Rev Urol.

[3] Worcester EM, Coe FL (2010). Clinical practice. Calcium kidney stones. N Engl J Med.

[4] Uribarri J, Oh MS, Carroll HJ (1989). The first kidney stone. Ann Intern Med.

[5] Saigal CS, Joyce G, Timilsina AR (2005). Direct and indirect costs of nephrolithiasis in an employed population: opportunity for disease management?. Kidney Int.

[6] Lotan Y, Cadeddu JA, Roerhborn CG, Pak CY, Pearle MS (2004). Cost-effectiveness of medical management strategies for nephrolithiasis. J Urol.

[7] Parks JH, Worcester EM, Coe FL, Evan AP, Lingeman JE (2004). Clinical implications of abundant calcium phosphate in routinely analyzed kidney stones. Kidney Int.

[8] Parks JH, Coe FL (1996). The financial effects of kidney stone prevention. Kidney Int.

[9] Mandel NS, Mandel GS (1989). Urinary tract stone disease in the United States veteran population. II. Geographical analysis of variations in composition. J Urol.

[10] Daudon M, Bouzidi H, Bazin D (2010). Composition and morphology of phosphate stones and their relation with etiology. Urol Res.

[11] Pak CY, Britton F, Peterson R, Ward D, Northcutt C, Breslau NA (1980). Ambulatory evaluation of nephrolithiasis. Classification, clinical presentation and diagnostic criteria. Am J Med.

[12] Worcester EM, Gillen DL, Evan AP, Parks JH, Wright K, Trumbore L (2007). Evidence that postprandial reduction of renal calcium reabsorption mediates hypercalciuria of patients with calcium nephrolithiasis. Am J Physiol Renal Physiol.

[13] Coe FL, Favus MJ, Crockett T, Strauss AL, Parks JH, Porat A (1982). Effects of low-calcium diet on urine calcium excretion, parathyroid function and serum 1,25(OH)2D3 levels in patients with idiopathic hypercalciuria and in normal subjects. Am J Med.

[14] Worcester EM, Coe FL (2008). New insights into the pathogenesis of idiopathic hypercalciuria. Semin Nephrol.

[15] Sakhaee K, Maalouf NM, Kumar R, Pasch A, Moe OW (2011). Nephrolithiasis-associated bone disease: pathogenesis and treatment options. Kidney Int.

[16] Turk C, Knoll T, Petrik A, European Association of U (2014). EUA Guidelines on Urolithiasis.

[17] Reid IR, Ames RW, Orr-Walker BJ, Clearwater JM, Horne AM, Evans MC (2000). Hydrochlorothiazide reduces loss of cortical bone in normal postmenopausal women: a randomized controlled trial. Am J Med.

[18] LaCroix AZ, Ott SM, Ichikawa L, Scholes D, Barlow WE (2000). Low-dose hydrochlorothiazide and preservation of bone mineral density in older adults: a randomized, double-blind, placebo-controlled trial. Ann Intern Med.

[19] van de Klift M, de Laet C, Herings R, Stijnen T, Pols H, Stricker B, et al. Thiazide diuretics and the risk for hip fracture. Ann Intern Med. 2003. 10.7326/0003-4819-139-6-200309160-00010 13679324

[20] Vaidya JS, Wenz F, Bulsara M, Tobias JS, Joseph DJ, Keshtgar M (2014). Risk-adapted targeted intraoperative radiotherapy versus whole-breast radiotherapy for breast cancer: 5-year results for local control and overall survival from the TARGIT-A randomised trial. Lancet.

[21] Borghi L, Meschi T, Guerra A, Novarini A (1993). Randomized prospective study of a nonthiazide diuretic, indapamide, in preventing calcium stone recurrences. J Cardiovasc Pharmacol.

[22] Ettinger B, Citron JT, Livermore B, Dolman LI (1988). Chlorthalidone reduces calcium oxalate calculous recurrence but magnesium hydroxide does not. J Urol.

[23] Laerum E, Larsen S (1984). Thiazide prophylaxis of urolithiasis. A double-blind study in general practice. Acta Med Scand.

[24] Mortensen JT, Schultz A, Ostergaard AH (1986). Thiazides in the prophylactic treatment of recurrent idiopathic kidney stones. Int Urol Nephrol.

[25] Ohkawa M, Tokunaga S, Nakashima T, Orito M, Hisazumi H (1992). Thiazide treatment for calcium urolithiasis in patients with idiopathic hypercalciuria. Br J Urol.

[26] Wilson DR, Strauss AL, Manuel MA. Comparison of medical treatments for the prevention of recurrent calcium nephrolithiasis. In: Kidney International; 1984: Blackwell Science Inc 350 Main St, Malden, Ma 02148; 1984. p. 994–994.

[27] Robertson WG, Peacock M, Selby PL, Williams RE, Clark P, Chisholm GD, et al. A multicentre trial to evaluate three treatments for recurrent idiopathic calcium stone disease—a preliminary report. In: Urolithiasis and related clinical research: Springer; 1985. p. 545–548.

[28] Fernández-Rodríguez A, Arrabal-Martín M, García-Ruiz M, Arrabal-Polo MA, Pichardo-Pichardo S, Zuluaga-Gómez A (2006). Papel de las tiazidas en la profilaxis de la litiasis cálcica recidivante. Actas Urológicas Españolas.

[29] Brocks P, Dahl C, Wolf H, Transbol I (1981). Do thiazides prevent recurrent idiopathic renal calcium stones?. Lancet.

[30] Ahlstrand C, Sandvall K, Tiselius HG (1996). Prophylactic treatment of calcium stone formers with hydrochlorothiazide and magnesium.

[31] Scholz D, Schwille PO, Sigel A (1982). Double-blind study with thiazide in recurrent calcium lithiasis. J Urol.

[32] Fink HA, Wilt TJ, Eidman KE, Garimella PS, MacDonald R, Rutks IR (2013). Medical management to prevent recurrent nephrolithiasis in adults: a systematic review for an American College of Physicians Clinical Guideline. Ann Intern Med.

[33] Reilly RF, Peixoto AJ, Desir GV (2010). The evidence-based use of thiazide diuretics in hypertension and nephrolithiasis. Clin J Am Soc Nephrol.

[34] Martins MC, Meyers AM, Whalley NA, Margolius LP, Buys ME (1996). Indapamide (Natrilix): the agent of choice in the treatment of recurrent renal calculi associated with idiopathic hypercalciuria. Br J Urol.

[35] Vigen R, Weideman RA, Reilly RF (2011). Thiazides diuretics in the treatment of nephrolithiasis: are we using them in an evidence-based fashion?. Int Urol Nephrol.

[36] Flack JM, Cushman WC (1996). Evidence for the efficacy of low-dose diuretic monotherapy. Am J Med.

[37] Huen SC, Goldfarb DS (2007). Adverse metabolic side effects of thiazides: implications for patients with calcium nephrolithiasis. J Urol.

[38] Pearle MS, Goldfarb DS, Assimos DG, Curhan G, Denu-Ciocca CJ, Matlaga BR (2014). Medical management of kidney stones: AUA guideline. J Urol.

